# Deciphering intratumoral heterogeneity of hepatocellular carcinoma with microvascular invasion with radiogenomic analysis

**DOI:** 10.1186/s12967-023-04586-6

**Published:** 2023-10-18

**Authors:** Yi Wang, Gui-Qi Zhu, Rui Yang, Cheng Wang, Wei-Feng Qu, Tian-Hao Chu, Zheng Tang, Chun Yang, Li Yang, Chang-Wu Zhou, Geng-Yun Miao, Wei-Ren Liu, Ying-Hong Shi, Meng-Su Zeng

**Affiliations:** 1 Department of Liver Surgery and Transplantation, Zhongshan Hospital, Liver Cancer Institute, Fudan University, 180 FengLin Road, Shanghai, 200032 China; 2 Department of Radiology, Zhongshan Hospital, Shanghai Institute of Medical Imaging, Fudan University, Xuhui District, Shanghai, 200032 China

**Keywords:** Hepatocellular carcinoma, Microvascular invasion, Radiomics, Single-cell RNA-seq

## Abstract

**Background and aims:**

The recurrence and metastasis of hepatocellular carcinoma (HCC) are mainly caused by microvascular invasion (MVI). Our study aimed to uncover the cellular atlas of MVI^+^ HCC and investigate the underlying immune infiltration patterns with radiomics features.

**Methods:**

Three MVI positive HCC and three MVI negative HCC samples were collected for single-cell RNA-seq analysis. 26 MVI positive HCC and 30 MVI negative HCC tissues were underwent bulk RNA-seq analysis. For radiomics analysis, radiomics features score (Radscore) were built using preoperative contrast MRI for MVI prediction and overall survival prediction. We deciphered the metabolism profiles of MVI^+^ HCC using scMetabolism and scFEA. The correlation of Radscore with the level of APOE^+^ macrophages and iCAFs was identified. Whole Exome Sequencing (WES) was applied to distinguish intrahepatic metastasis (IM) and multicentric occurrence (MO). Transcriptome profiles were compared between IM and MO.

**Results:**

Elevated levels of APOE+ macrophages and iCAFs were detected in MVI^+^ HCC. There was a strong correlation between the infiltration of APOE^+^ macrophages and iCAFs, as confirmed by immunofluorescent staining. MVI positive tumors exhibited increased lipid metabolism, which was attributed to the increased presence of APOE+ macrophages. APOE^+^ macrophages and iCAFs were also found in high levels in IM, as opposed to MO. The difference of infiltration level and Radscore between two nodules in IM was relatively small. Furthermore, we developed Radscore for predicting MVI and HCC prognostication that were also able to predict the level of infiltration of APOE^+^ macrophages and iCAFs.

**Conclusion:**

This study demonstrated the interactions of cell subpopulations and distinct metabolism profiles in MVI^+^ HCC. Besides, MVI prediction Radscore and MVI prognostic Radscore were highly correlated with the infiltration of APOE^+^ macrophages and iCAFs, which helped to understand the biological significance of radiomics and optimize treatment strategy for MVI^+^ HCC.

**Supplementary Information:**

The online version contains supplementary material available at 10.1186/s12967-023-04586-6.

## Introduction

Hepatocellular carcinoma (HCC) as a vascular rich tumor with dual blood supply characteristics, is highly susceptible to microvascular invasion (MVI) [1, 2]. The reported incidence of MVI in HCC is 30–60%, which is related to clinical staging and pathological characteristics [3, 4]. MVI is the most important pathological mechanism leading to recurrence and metastasis of HCC. Invasion of microvessels indicates the trend of tumor metastasis, and microvessels become the first stop of metastasis. The three stages of tumor thrombus development include MVI, portal vein invasion, and intrahepatic and extrahepatic metastasis. Therefore, MVI is a process in which the malignancy of tumor cells gradually increases and destroys the surrounding tissue structure [5]. From the cellular change level, the initiation of MVI is correlated with epithelial–mesenchymal transition (EMT) [6]. During this process, tumor microenvironment (TME) remodeling might also play an important role in mediating HCC invasion. Lu et al. found MMP9^+^ macrophages promoting HCC invasion were more infiltrated in metastatic HCC [7]. Cancer-associated fibroblasts (CAFs) secrete cytokines, growth factors, and metabolites that affect the behavior and function of tumor cells and other matrix components to promote metastasis [8]. Liu et al. reported CAFs secret CCL5, CCL7, CCL2 and CXCL16 promoting HCC metastasis by activating TGF-β pathways [9]. Moreover, abundant evidences show that the interplay among tumor cells, immune cells and stromal cells contributes to the metastasis of HCC [10]. Deciphering the tumor environment of HCC with MVI using single-cell RNA sequencing (scRNA-seq) can be helpful for developing potentially effective immunotherapy strategies.

Preoperative diagnosis of MVI in HCC patients has significant clinical value in establishing ablation surgery plan. For MVI positive patients, ablation margin > 1 cm is suggested [11]. Therefore, many studies focused on the imaging features and laboratory tests to predict MVI and demonstrated good prediction effects. Radiomic analysis can comprehensively reflect the heterogeneity of tumor. Yang et al. constructed preoperative radiomics nomogram to predict MVI of HCC and exhibited C-index of 0.936 and 0.864 in the training and validation cohort [12]. Deciphering the biological context of radiomic features can promote their generalization in clinical use. Radiogenomic analysis correlated radiomic analysis with genomics or transcriptomics, having the potential to evaluate tumor microenvironment noninvasively [13, 14]. However, there is scarce data of exploring the relationship of radiomic features with TME of HCC.

Therefore, in the present study, we dissected TME of HCC with MVI utilizing multidimensional data including bulk-level RNA sequencing (RNA-seq) and scRNA-seq. Moreover, we also investigated the heterogeneity of intrahepatic metastasis (IM) and multicentric occurrence (MO) of HCC using whole exome sequencing (WES) and bulk-level RNA-seq. Radiomics data was integrated to illustrate the biological correlation of radiomic features with TME.

## Materials and methods

We retrospectively recruited HCC patients whose baseline dynamic contrast-enhanced MRI were suitable for radiomics analysis from three independent data sets. All analyses were approved by the institutional review board of Zhongshan Hospital, Fudan University, Affiliated Hospital of Nantong University and Zhongshan Hospital Fudan University Xiamen Branch. All analyses were conducted according to the Declaration of Helsinki. Due to the retrospective nature of the study, and full anonymization of data, written informed consent were waived from patients.

### Patient cohort preparation

**Fig. 1:**
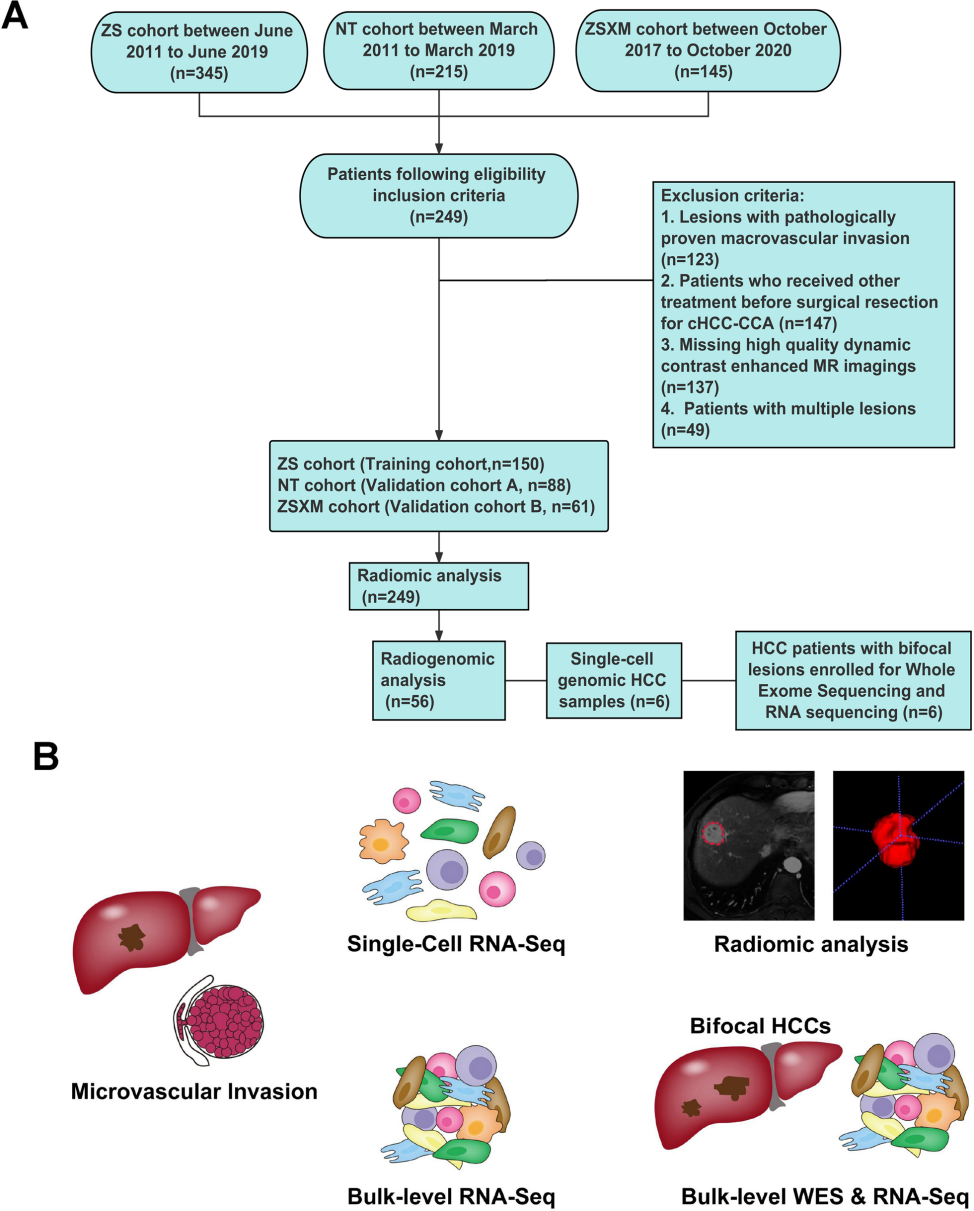
Study flowchart of this radiogenomic study. **A** Description of the radiomic cohorts, RNA-seq cohort, WES samples and single-cell RNA-seq samples. **B** Framework of this integrative radiogenomic analysis

Our study included cohorts of HCC patients from Zhongshan Hospital of Fudan Univeristy. 56 HCC patients with RNA-seq data, 6 patients of 12 HCC nodules with WES data and 6 patients with single-cell RNA-seq data. For radiomics analysis, we identified consecutive HCC patients who underwent surgical ablation from three separate datasets. A training dataset from Zhongshan Hospital of Fudan Univeristy (ZS cohort, a total of 150 patients), an external dataset from Affiliated Hospital of Nantong University (NT cohort, a total of 88 patients) and another external dataset from Zhongshan Hospital Fudan University Xiamen Branch (ZSXM cohort, a total of 61 patients). The patient enrollment process was presented in Fig 1.

### MRI protocol, imaging analysis and radiomics feature extraction

Manual tumor segmentation using ITK-SNAP software was performed by two blinded radiologists (C.Y. and C.W.Z.) with 16 and 13 years of experience interpreting abdomen MRI scans. The clinical and pathological data of HCC patients were not disclosed. The radiomics features extraction was conducted by the “PyRadiomics” R package. To assess reproducibility, 100 patients were randomly selected from the entire group to evaluate the consistency of regions of interest by the two radiologists using the intra-class correlation coefficient (ICC). Subsequently, 2320 radiomics features were extracted for each patient from the tumor in arterial, portal venous, and delayed phases. The radiomics features consisted of 32 non-texture characteristics, such as shape, size, and first-order statistics, as well as 75 texture features, including Grey Level Co-occurrence Matrix (GLCM), Grey Level Run Length Matrix Features (GLRLM), Gray Level Size Zone Matrix (GLSZM), Neighboring Gray Tone Difference Matrix (NGTDM), and Gray Level Dependence Matrix (GLDM). The values of the radiomics features were standardized using the z-score method.

Patients in ZS cohort and ZSXM cohort were both scanned with 1.5T MR scanner (uMR 560, United Imaging Healthcare). Patients in NT cohort were scanned with 1.5 T MR scanner (MAGNETOM Aera, Siemens Healthcare). Routine liver protocols consisted of transverse T2-weighted imaging (T2WI), T1WI, in-phase and opposed-phase sequences, and diffusion-weighted imaging (DWI, b value = 0, 50, and 500 s/mm^2^).

### Establishment and validation of prognostic radiomics model and MVI prediction radiomics model

We employed a two-step approach in selecting radiomics features for prognosis. The first step involved utilizing univariate Cox regression analysis to eliminate radiomics features with a false discovery rate (FDR) below 0.05. In the second step, the radiomics features selected in the initial step were subjected to least absolute shrinkage and selection operator (LASSO) regression using the “glmnet” R package, with the “Cox” family set as the algorithm. The coefficients assigned to the final selected radiomics features were utilized to calculate the prognostic radiomics score (Radscore) for each patient. To determine the optimal cutoff value for categorizing patients into low-risk and high-risk groups, we utilized the “surv_cutpoint” function provided by the “survminer” R package based on the maximal log-rank test. We compared the differences in overall survival (OS) using the log-rank test. Kaplan–Meier analysis was employed to evaluate the correlation between survival and prognostic Radscore.

For constructing MVI prediction radiomics model, we used tenfold cross validation LASSO model to select MVI associated radiomics features in the training cohort. Then we used logistic regression to develop MVI prediction signature. Receiver operating characteristic (ROC) curves were plotted to assess the predictive value of MVI prediction Radscore.

### Bulk-level RNA-seq analysis

Total RNA was extracted and purified from 56 HCC fresh frozen tissues using the Whole RNA extraction kit. Sufficient quantities (1–2 μg) of high-quality (RNA integrity > 8), DNA free RNA samples were used for library construction. cDNA synthesis, end repair, poly-A tail, and addition of splice sequences were carried out according to the TruSeqRNA Library Prep Kit instructions. The successfully constructed cDNA library was sequenced on the Illumina NovaSeq 6000 platform for transcriptome. Fragments Per Kilobase of exon model per Million mapped fragments (FPKM) values were obtained using the tophat-cufflinks pipeline. Fastq files were mapped to human reference genome hg19 (UCSC human reference genome hg19).

### Bulk whole-exome sequencing and data processing

1. Extraction: DNA is extracted from tissue samples using a self-prepared reagent using salting out method, which increases salt concentration to reduce protein solubility and promote aggregation and precipitation. Mix STE, SDS, and protease K into a centrifuge tube with tissue and incubate in a constant temperature water bath for 3 days. During this period, continuously add protease K until the liquid in the centrifuge tube is transparent and the tissue is completely digested. After complete digestion of the tissue, add NaCl solution separately, place at − 20 ℃ for 10 min, centrifuge at 15,000 r/min, retain the supernatant and discard the precipitate. Repeat the operation once. Afterwards, approximately twice the volume of pre-cooled anhydrous ethanol is added to the centrifuge tube, and DNA is precipitated at − 20 ℃ for 15 min. The supernatant is then centrifuged at 15,000 r/min and discarded. Then add pre-cooled 70% ethanol for washing, centrifuge at 15,000 r/min, discard the supernatant, and dry at room temperature. Finally, add TE to dissolve DNA and store at -20 ℃.
2. Quality control: use Agilent2100 biological analyzer to detect DNA integrity. Separate DNA fragments of different sizes from the sample using capillary electrophoresis and embed fluorescent dyes to quantify DNA concentration based on fluorescence signal intensity.
3. Whole exome sequencing: pooling the library according to its effective concentration and target data volume requirements before conducting sequencing. The sequencing platform is Illumina NovaSeq 6000, which generates pair end sequencing data with a sequencing read length of 2 × 150 bp, Q30 ≥ 80% is qualified data. In this study, a total of 12 tumor samples were sequenced at a depth of 200× for each tumor sample library, while 6 adjacent cancer samples were sequenced at a depth of 100× for each adjacent cancer sample library.
4. Sequencing data quality control: the data from the sequencer after off machine is called raw data. Fastp software is used to remove connector contamination and low-quality data, and the original sequencing data is filtered to get clean reads. The filtering criteria include: first, removing the joint sequence; Afterwards, remove no less than 5 reads of N bases (i.e. non AGCT bases); Using a sliding window of 4 bases as a unit, remove reads with an average base mass value less than 20; After the above filtering, remove reads with a length of less than 75 bp or an average base mass value of less than 15.
5. Reference genome alignment: use BWA software to compare the filtered clean reads to the reference genome (the reference genome version is human_glk_v37). Format the comparison results using SAMtools software, and then use Picard (http://broadinstitute.github.io/card/) software to remove PCR duplicates.
6. Mutation detection analysis: firstly, use the BaseCalibrator module of GATK4 software to recalibrate the base quality to improve the accuracy of mutation detection. Afterwards, the Haplotypecaller module of GATK4 software was used to detect single nucleotide polymorphisms (SNP) and insertion and deletion (InDel) in the samples, and a QD ≥ 2.0 standard was used for filtering to reduce the error rate of SNP and InDel detection. Afterwards, Anovar software was used to annotate the detected SNP and InDel into databases such as Refseq, Thousand Genomes, EXACm, and COSMIC.
7. Detection of somatic mutation: for tumor samples and matched normal samples, MuTect2 software is used to detect single nucleotide variant (SNV) and InDel sites in somatic cell cells, and Anovar software is used for annotation.

### scRNA-seq data processing

#### Dimension reduction and clustering analysis

We converted the merged matrix into a seurat object for subsequent analysis using the function ‘CreateSeuratObject’. The expression matrix was standardized by using the function ‘NormalizeData’. Then we detected the top 1500 genes with the highest variation in the expression matrix using the function ‘FindVariableFeatures’. Based on these 1500 genes, we used the function ‘ScaleData’ to normalize the matrix. Subsequently, the dimensionality of the expression matrix was reduced through the ‘RunPC’ function. After completing the preliminary PCA dimensionality reduction, we used the function ‘RunHarmony’ to de batch the single cell expression matrixes from different samples, and selected appropriate ‘Harmony’ parameters through the ‘Embedding’ function. Use the ‘RunUMAP’ function to further reduce the dimensionality of the ‘harmony’ value in the seurat object. Then we completed cluster analysis through functions ‘FindNeighbors’ and ‘FindClusters’. Same procedures were conducted for sub-clustering. We defined distinct cell subtypes in HCC based on the gene signatures of each cell type and known lineage markers. We visualized cell subtypes on a 2D map produced with t-distributed stochastic neighbour embedding (t-SNE).

#### Differential expression and pathway analysis

We used ‘FindMarkers’ function to perform differential gene expression analysis on different cell populations and set Padj = 0.05 as the cut-off value. Next, we conducted pathway enrichment analysis on these differentially expressed genes using cluster profiler (V3.19.0). We set P = 0.05 as the cut-off value.

#### Receptor-ligand communication between cell types

CellChat (1.1.2) was applied to investigate cell-to-cell interaction among different cell types. ‘CellChatDB. human’ was utilized as the receptor-ligand interaction database.

When evaluating the regulatory network of APOE^+^ macrophages on iCAFs, iCAFs were considered as reference receiver cells to check the regulatory potential of APOE^+^ macrophages.

#### Gene regulatory network inference

We analyzed the enrichment of transcriptome factors in APOE^+^ macrophages using SCENIC (v1.1.0) with default settings. The input matrices for each sample in SCENIC were the raw UMI counts from Seurat.

### Evaluation of metabolic activity

The metabolic profiles were compared between different cells through scMetabolism by calculating pathways quantification using ssGSEA [15]. scFEA was applied to evaluate metabolite abundance based on scRNA-seq data [16]. Gene set variation analysis (GSVA) was applied to calculate the enrichment score of each metabolic pathway in each sample with transcriptomic data.

### Estimation of immune cells infiltration in bulk RNA-seq cohort

CIBERSORTx was used to create a reference signature matrix from our scRNA-seq dataset and estimate cell subsets proportions from our RNA-seq dataset based on constructed cell-type reference.

### Multiplex immunohistochemistry

Multiplex staining was performed using TSA 7-color kit (D110071-50T, Yuanxibio), according to manufacturer’s instruction. The panel was PDGFRA (Cat# ab252922, Abcam), APOE (Cat #ab7817, Abcam), CD68 (Cat #97778, CST). Primary antibodies were sequentially applied, followed by horseradish peroxidase-conjugated secondary antibody incubation (1:1, Cat# DS9800, Lecia Biosystems; 1:1 Cat# A10011-6/A10012-6, WiSee Biotechnology), and tyramide signal amplification (M-D110051, WiSee Biotechnology). The slides were microwave heat-treated after each TSA operation. Nuclei were stained with DAPI (D1306, ThermoFisher) after all the antigens above being labeled. The stained slides were scanned to obtain multispectral images using the Pannoramic MIDI imaging system (3D HISTECH).

### Co-culture experiments

Co-culture experiments were performed by seeding PMA-differentiated THP1-derived macrophages in the lower chamber and Huh7 cells in the upper chamber of a 6 well transwell apparatus with 0.4 μm pore size. The co-cultured THP1-derived macrophages were subjected to further analysis after 48 h of co-culture.

### Quantitative real-time PCR (qRT-PCR)

Total RNA was extracted from THP1-derived macrophages and Huh7 cells using TRIzol as recommended by the manufacturer’s protocol. RNA was reversely transcribed using oligo-Dt primers. Diluted cDNA was then used in qRT-PCR reactions containing SYBR Premix ExTaq and gene-specific primers. The reactions were performed in a QuantStudioTM 6 Flex Real-Time PCR System. β-Actin was used as the internal reference gene. Primers used in this study are listed in Additional file 1: Table S1.

### Lentivirus vector construction and transfection

For gene silencing, THP1 were transduced with PLKO.1 lentivirus carrying target gene-specific shRNA constructs. Lentivirus was provided by Gene Pharma Inc. (Shanghai, China). The multiplicity of infection [MOI] of 40 was used to infect THP1. qRT-PCR were conducted to check the transfection efficiency.

### Statistical analysis

Kaplan–Meier method was used to construct survival curves and log-rank test was used to compare the survival differences. Multivariate Cox proportional hazard regression model for screening prognostic covariates and calculate hazard ratios (HRs) and 95% confidence intervals. Spearman’s correlation was applied to conducted correlation analysis. Student’s t-test was used to compared continuous variables with normally distributed variables. Mann-Whitney U-test was used to compare continuous variables with non-normally distributed variables. Two-sided p-values less than 0.05 were considered statistically significant. All statistical analyses were performed with R software.

## Results

### Tumor-specific APOE^+^ macrophages are associated with MVI of HCC

**Fig. 2:**
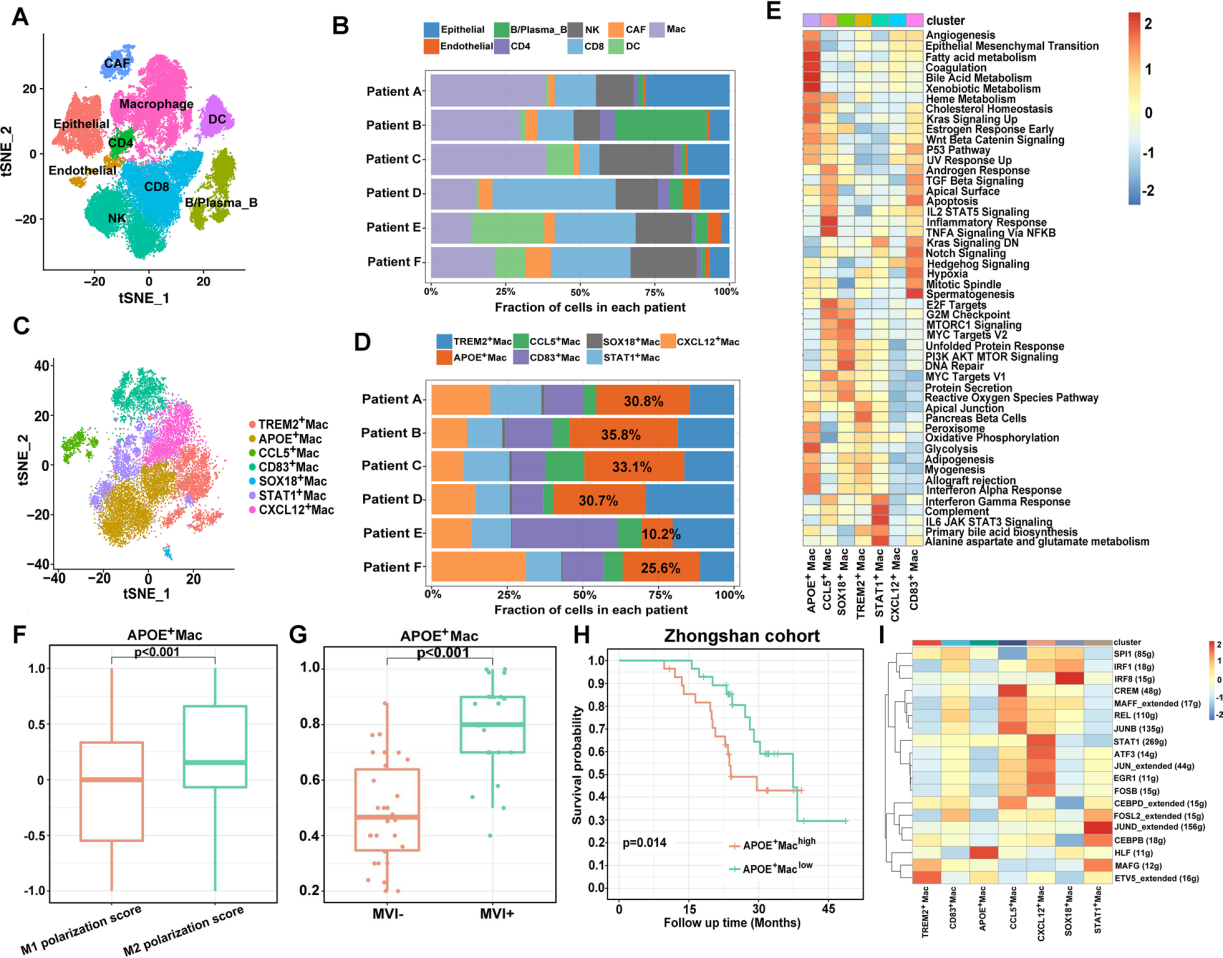
scRNA-seq profiling of multicellular ecosystem in MVI^+^ HCC and MVI^−^ HCC. **A** tSNE plot of cells from MVI^+^ HCC and cells from MVI^−^ HCC of 6 HCC patients with 9 clusters. **B** Proportion of 9 major cell types showing in bar plots in different samples. **C** tSNE showing the composition of macrophages. **D** Bar plots showing the percentage of each macrophage subtypes in scRNA-seq. **E** GSVA of 7 subclusters of macrophages. **F** The distribution of M1 and M2 polarization score in APOE^+^ macrophages. **G** The infiltration of APOE^+^ macrophages calculated by CIBERSORTX in MVI^+^ HCC and MVI^−^ HCC samples. **H** The Kaplan–Meier overall survival curves of HCC patients stratified by APOE^+^ macrophages infiltration. **I** Heatmap showing significantly different transcription factors genes in each macrophages subtype

We recruited 3 MVI^+^ HCC (Patient A, Patient B, and Patient C) and 3 MVI^-^ HCC patients (Patient D, Patient E, and Patient F) in our institution. Patient A and Patient B were classified into BCLC 0 stage. Patient C, Patient D, Patient E and Patient F were classified into BCLC A stage. Besides, 56 HCC patients undergoing bulk RNA-seq were enrolled in the study, the baseline characteristics were displayed in Table 1. We merged the scRNA-seq data across all the samples. After stringent quality control, we obtained single-cell transcriptomes for 55,525 single cells, in which cells were originated from MVI^+^ HCC tissues and MVI^-^ HCC tissues. All samples were integrated with Harmony algorithm to modify the batch effect. Dimensionality reduction and unsupervised clustering analysis were performed. Subsequently, the cells were categorized into nine major cell types (Fig. 2A), including macrophages (n = 14,298, marked with CD163 and CD68), epithelial cells (n = 6141, marked with EPCAM and CDH1), CD8^+^ T cells (n = 12,305, identified by CD3D and CD8A), CD4^+^ T cells (n = 1682, identified by CD3D and CD4), dendric cells (DCs) (n = 3429, identified by ITGAX), endothelial cells (n = 1489, marked with PECAM1 and vWF), natural killer (NK) cells (n = 8630, marked with FGFBP2 and FCG3RA), B/Plasma_B (n = 5392, marked with CD19, JCHAIN and CD79A) and cancer-associated fibroblasts (CAFs) (n = 2159, marked with ACTA2 and COL1A2). The abundance of infiltration of each cell type was variable in each patient, indicating the heterogeneity in the progression of HCC progression (Fig. 2B). For example, macrophages were remarkably higher infiltrated in MVI^+^ HCC samples while CD8^+^ T cells were higher infiltrated in the MVI^-^ HCC samples.

**Table 1:** Baseline characteristics of 56 HCC patients in bulk RNA-seq cohort

	MVI−	MVI+	P value
Age	57.73 ± 11.05	61.80 ± 11.14	0.177
Sex (male)	15 (50.0%)	19 (73.1%)	0.667
AFP (> 400 ng/mL)	21 (70.0%)	11 (42.3%)	0.001
BCLC (A)	21 (70.0%)	11 (42.3%)	0.001
Recurrence	17 (65.4%)	11 (42.3%)	0.032

Data were compared using the chi-square test or t test

*AFP* α-fetoprotein, *BCLC* Barcelona Clinic Liver Cancer

We speculated that macrophages played important roles in the initiation of MVI of HCC. Hence, we focused on macrophages to assess the distinct subsets infiltrated in the MVI^+^ HCC and MVI^-^ HCC tissues. Macrophages were clustered into 7 subpopulations based on their distinct transcriptomic signatures (Fig. 2C). We observed that APOE^+^ macrophages were significantly enriched in MVI^+^ HCC samples compared with MVI^−^ HCC samples (Fig. 2D). GSVA showed that APOE^+^ macrophages showed an increased level of lipid-associated metabolism, angiogenesis and epithelial mesenchymal transition (Fig. 2E). By calculating M1/M2 polarization scores, we found that APOE^+^ macrophages skewed towards M2 polarization (Fig. 2F). We utilized CIBERSORTx to predict the abundance of cell types calculated by scRNA-seq in our RNA-seq cohort containing 26 MVI^+^ HCC and 30 MVI^−^ HCC tissues. We found that greater infiltration of APOE^+^ macrophages was associated with MVI (p < 0.01, Fig. 2H) and worse OS (log-rank test, p = 0.014, Fig. 2G).

T cells play an indispensable role in the immune microenvironment. We identified 6 main groups, including 3 clusters of CD8^+^ T cells and 3 clusters of CD4^+^ T cells based on gene expression signatures. The 6 subtypes were CD8_Naive T cells, CD8_Effector T cells, CD8_Exhausted T cells, CD4_Treg, CD4_Memory T cells and CD4_Effector T cells. In MVI^+^ HCC samples, CD8_Exhausted T cells and CD4_Treg were identified higher infiltrated than in MVI^−^ HCC samples. In addition, a greater number of CD8_Effector T cells infiltrated in MVI^+^ HCC than MVI^−^ HCC samples (Additional file 1: Fig. S1A–D). The high correlation of APOE^+^ macrophages with CD8_Effector T cells and CD4_Treg was observed in ZS cohort (Additional file 1: Fig. S1E, F).

To identify the master regulator of APOE^+^ macrophages, SCENIC analysis indicated that hepatic leukaemia factor (HLF), a transcription factor associated with tumorigenesis, immune functions and metabolism [17, 18], was highly active in APOE^+^ macrophages (Fig. 2I). In our cohort, HLF was positively correlated with fatty acid metabolism calculated by GSVA (Additional file 1: Fig. S2A) and infiltration of CD4_Treg (Additional file 1: Fig. S2B).

Next, we attempted to study how APOE^+^ macrophages enhanced MVI of tumor cells. After verifying the knock-down (KD) efficiency of short hairpin RNA (Additional file 1: Fig. S3A), we found that KD of APOE in THP-1 cells led to a dramatic increase of CDH1 whose expression negatively regulated epithelial mesenchymal transition (EMT). In addition, other metastasis related genes (VEGFA, MMP-2 and MMP-9) decreased in tumor cells (Additional file 1: Fig. S3B–E).

Together, these findings indicate that the active immune function of T cells is impaired in the TME of HCC with MVI. Moreover, APOE^+^ macrophages might function as an immune suppressive role in TME and promote the MVI of tumor cells.

### APOE^+^ macrophages and iCAFs interaction may contribute to the MVI of HCC

**Fig. 3:**
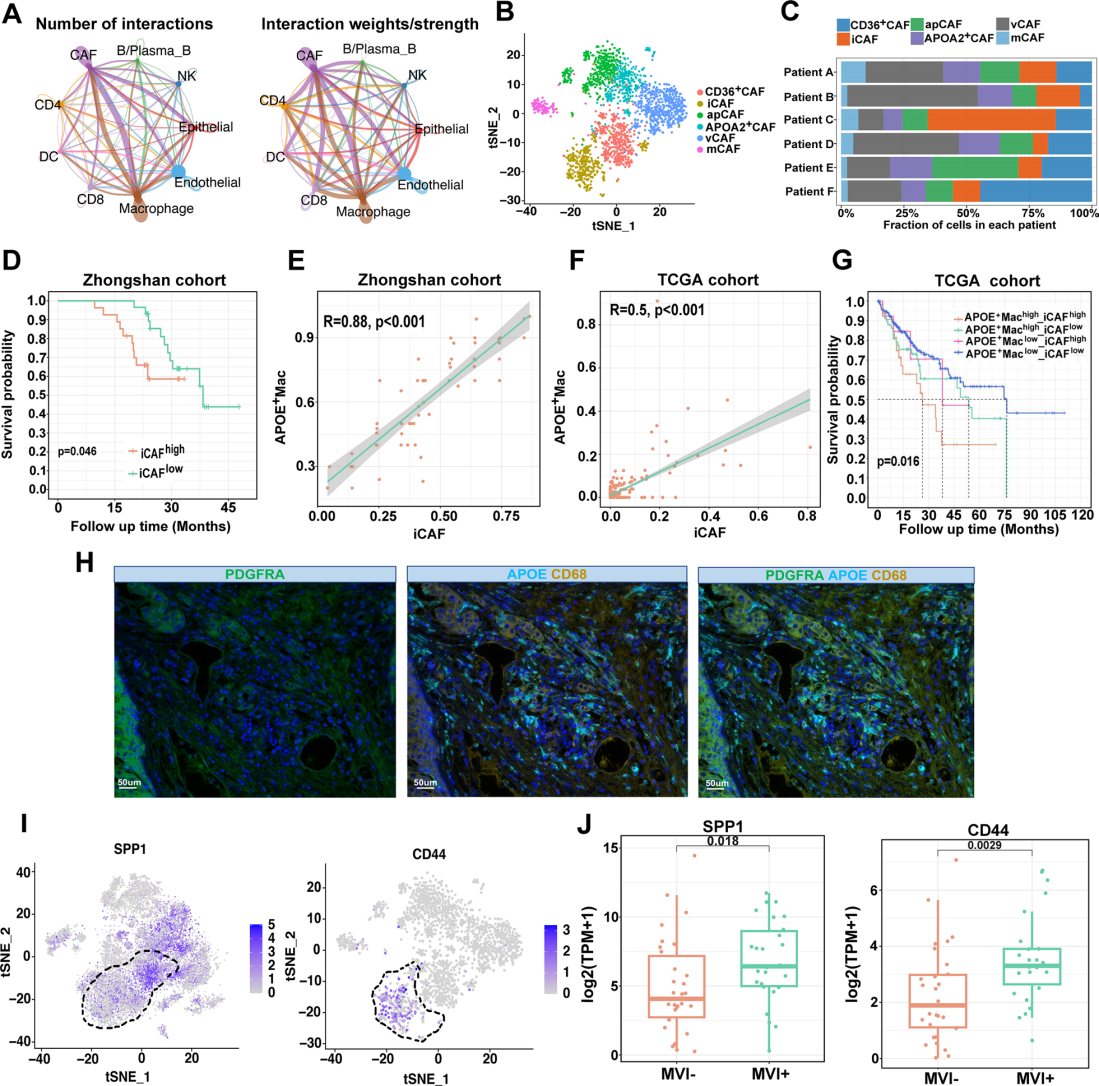
The interaction network between APOE^+^ macrophages and iCAFs. **A** Cell–cell communications between main nine cell types by Cell chat analysis. **B** tSNE showing the composition of iCAFs. **C** Proportion of 5 major cell types showing in bar plots in different samples. **D** The Kaplan–Meier overall survival curves of HCC patients stratified by iCAFs infiltration. **E**, **F** Scatter plots showing the correlation between the infiltration of APOE^+^ macrophages and iCAFs in Zhongshan cohort and TCGA cohort. **G** The Kaplan–Meier overall survival curves of HCC patients stratified by the infiltration of both APOE^+^ macrophages and iCAFs. **H** Representative IF staining of human HCC tissue with MVI. PDGFRA (green), APOE (blue), CD68 (gold). Bar, 50 μm. **I** tSNE plot showing expression levels of SPP1 and CD44 in HCC samples. **J** The comparison of expression level of SPP1 and CD44 in MVI^−^ HCC and MVI^+^ HCC

Cell-chat analysis presented diverse interactions among these nine cell types. The interaction between macrophages and CAFs were the most prominent (Fig. 3A). Subsequently, we deciphered the subclusters of CAFs and six main subpopulations were determined based on the expression of specific cellular markers: inflammatory CAFs (iCAFs) (marked by PDGFRA, IL-6, IL-11, CXCL1, and CXCL2), matrix CAFs (mCAFs) (marked by α-SMA and COL1A1), antigen-presenting CAFs (apCAFs) (marked by CD74 and HLA-DRA), vascular CAFs (vCAFs) (marked by MCAM, MYH1, and MUSTN1) CD36^+^ CAF and APOA2^+^ CAF (Fig. 3B). When observing the distribution of subclusters of CAFs in each patient, we found that iCAFs were enriched in the MVI^+^ HCC patients (Fig. 3C). High level of iCAFs were associated with worse OS in our cohort, indicating that iCAFs may be involved in the progression of HCC (Fig. 3D). Next, we attempted to investigate whether APOE^+^ macrophages and iCAFs have mutual effect. We found that the high correlation of APOE^+^ macrophages and iCAFs were observed in ZS cohort (R = 0.88, p < 0.001) and TCGA cohort (R = 0.5. p < 0.001) (Fig. 3E, F). Patients with both high APOE^+^ macrophages and iCAFs exhibited the shortest OS compared with other groups (p = 0.016), suggesting the synergistic effect of these two cell types can promote the MVI of HCC (Fig. 3G). Immunofluorescent labeling demonstrated the close proximity of PDGRA^+^ cells and APOE^+^CD68^+^ cells in HCC with MVI tissue (Fig. 3H). An increased intercellular interaction in SPP1-CD44 was observed between APOE^+^ macrophages and iCAFs in HCC patients with MVI (Additional file 1: Fig. S4). We found that APOE^+^ macrophages and iCAFs showed high expression of SPP1 and CD44, respectively (Fig. 3I). Then the expression level of SPP1 and CD44 were determined in HCC patients of our cohort. The results suggested that the expression level of SPP1 and CD44 were both higher in MVI^+^ HCC group (Fig. 3J).

### Metabolic heterogeneity between MVI^+^ HCC and MVI^-^ HCC

**Fig. 4:**
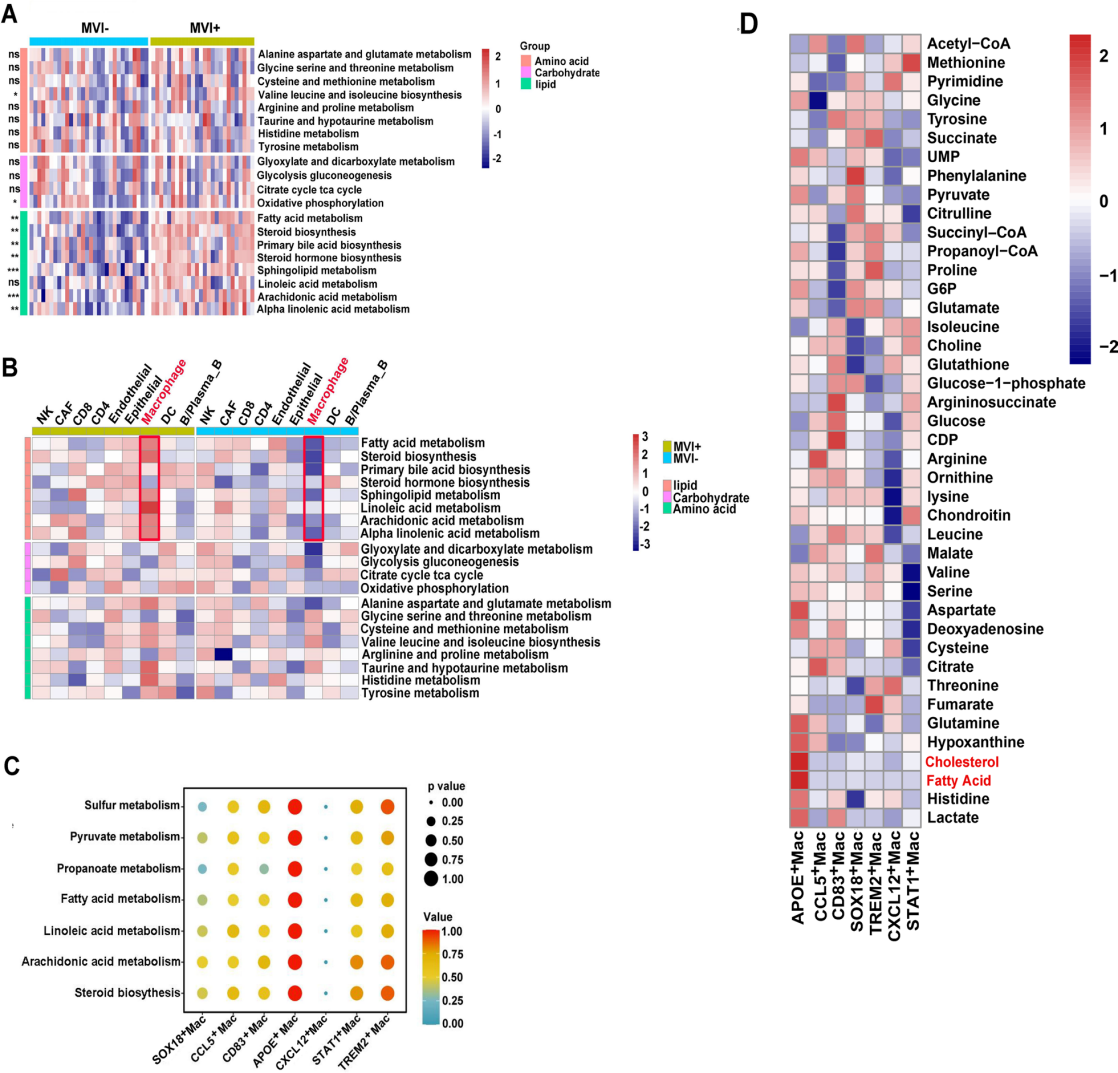
Comparison of metabolism landscape of MVI^+^ HCC and MVI^−^ HCC. **A** Heatmap of the three main categories of metabolism pathways for MVI^−^ HCC and MVI^+^ HCC using RNA-seq cohort. **B** Heatmap of the three main categories of metabolism pathways for nine cell types in scRNA-seq. **C** Lipid associated metabolism of seven subpopulations of macrophages. **D** Metabolite abundance of seven clusters of macrophages

Aberrant metabolism played major role in tumor development and metastasis. Hence, we compared the metabolic pathway activity between MVI^+^ HCC and MVI^−^ HCC utilizing GSVA. Lipid-associated metabolism pathways were significantly enriched in MVI positive patients, compared with amino acid and carbohydrate pathways (Fig. 4A). To investigate which cell type dominates such metabolic pattern of HCC with MVI, we used ScMetabolism to quantify scores of metabolic pathways including lipid, carbohydrate and amino acid among the nine cell types. We found that macrophages had higher activity of lipid-associated metabolism in HCC with MVI (Fig. 4B). Furthermore, among seven subpopulations of macrophages, APOE^+^ macrophages presented remarkably higher infiltration of lipid-associated metabolism (Fig. 4C). We also applied scFEA to calculate metabolite abundance of different clusters of macrophages. Fatty acid and cholesterol were distinctly enriched in APOE^+^ macrophages (Fig. 4D). The above results indicated that HCC with MVI was characterized with abnormally increased lipid-associated metabolism of APOE^+^ macrophages.

### Radiomics score was in close correlation with APOE^+^ macrophages and iCAFs

We explored whether radiomic features could predict MVI of HCC and have prognostic value. 150 patients from ZS cohort, 88 patients from NT cohort and 61 patients from ZSXM cohort were enrolled in our study. Six radiomic features were finally selected for identifying MVI (Additional file 1: Table S2). Identifying MVI yielded the AUCs of 0.857, 0.684 and 0.780 in the training cohort, validation cohort A and validation cohort B, respectively (Additional file 1: Fig. S5A). Risk prediction model was built based on the 16 prognostic radiomics features (Additional file 1: Table S4). The high Radscore was identified significantly associated with worse OS of HCC in the training cohort (p < 0.001), validation A cohort (p < 0.001) and validation B cohort (p < 0.001) (Additional file 1: Fig. S5B). The multivariate Cox proportional hazards model revealed that high level of APOE^+^ macrophages and high prognostic Radscore independently predicted worse OS in HCC patients (Table 2).

**Table 2:** Multivariate Cox proportional hazard model for OS in HCC radiogenomic cohort

Variables	OS	
	HR (95% CI)	P value
Age	0.99 (0.97–1.03)	0.84
AFP		
< 400 ng/mL	Ref.	
> 400 ng/mL	0.61 (0.27–1.38)	0.24
MVI		
MVI−	Ref.	
MVI+	1.88 (0.60–5.87)	0.03
BCLC		
0	Ref.	
A	0.40 (0.12–1.32)	0.13
Prognostic Radscore		
Low	Ref.	
High	2.38 (1.87–8.26)	0.02
APOE^+^ Mac		
Low	Ref.	
High	4.31 (1.49–12.45)	0.008
iCAF		
Low	Ref.	
High	1.34 (0.54–3.33)	0.52

*AFP* α-fetoprotein, *MVI* microvascular invasion, *BCLC* Barcelona Clinic Liver Cancer

**Fig. 5:**
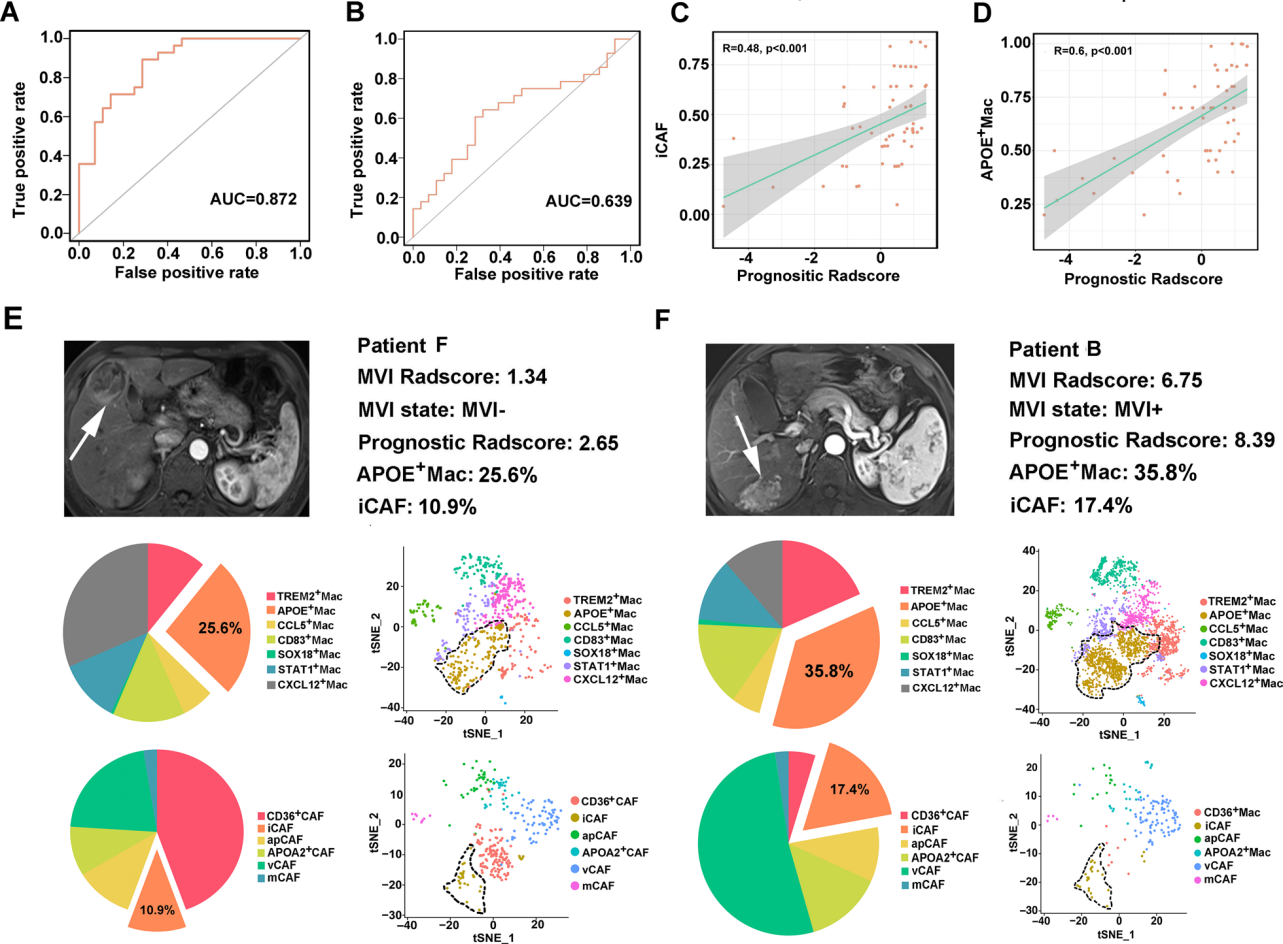
Radiomics score correlating with APOE^+^ macrophages and iCAFs. **A**, **B** AUC of the radiomic signature for predicting MVI in the training cohort and validation cohort. **C**, **D** Scatter plots showing the correlation between the infiltration of prognostic Radscore and iCAFs and APOE^+^ macrophages. **E**, **F** Pie charts showing the distribution of identified subclusters of macrophages and CAFs between different patients

Furthermore, we found that MVI prediction Radscore could also identify the level of APOE^+^ macrophages and iCAFs with satisfactory efficacy (AUC = 0.872 and 0.639, respectively, Fig. 5A, B). High prognostic Radscore was correlated with high level of iCAF (R = 0.48, p < 0.001) (Fig. 5C) and APOE^+^ macrophages (R = 0.6, p < 0.001) (Fig. 5D). We compared the tumor microenvironment component of patient F (MVI negative) and patient B (MVI positive) by scRNA-seq data. Patient B had higher abundance of APOE^+^ macrophages and iCAFs than patient F (33.3% vs. 19.5%, 15.2% vs. 9.4%, respectively). MVI prediction Radscore and prognostic Radscore were higher in Patient B than in Patient F (Fig. 5E, Additional file 1: Table S3). These data suggested that radiomics might be a promising approach for identifying the component of tumor microenvironment.

### Distinct transcriptome profiles and radiomics scores between IM and MO

**Fig. 6:**
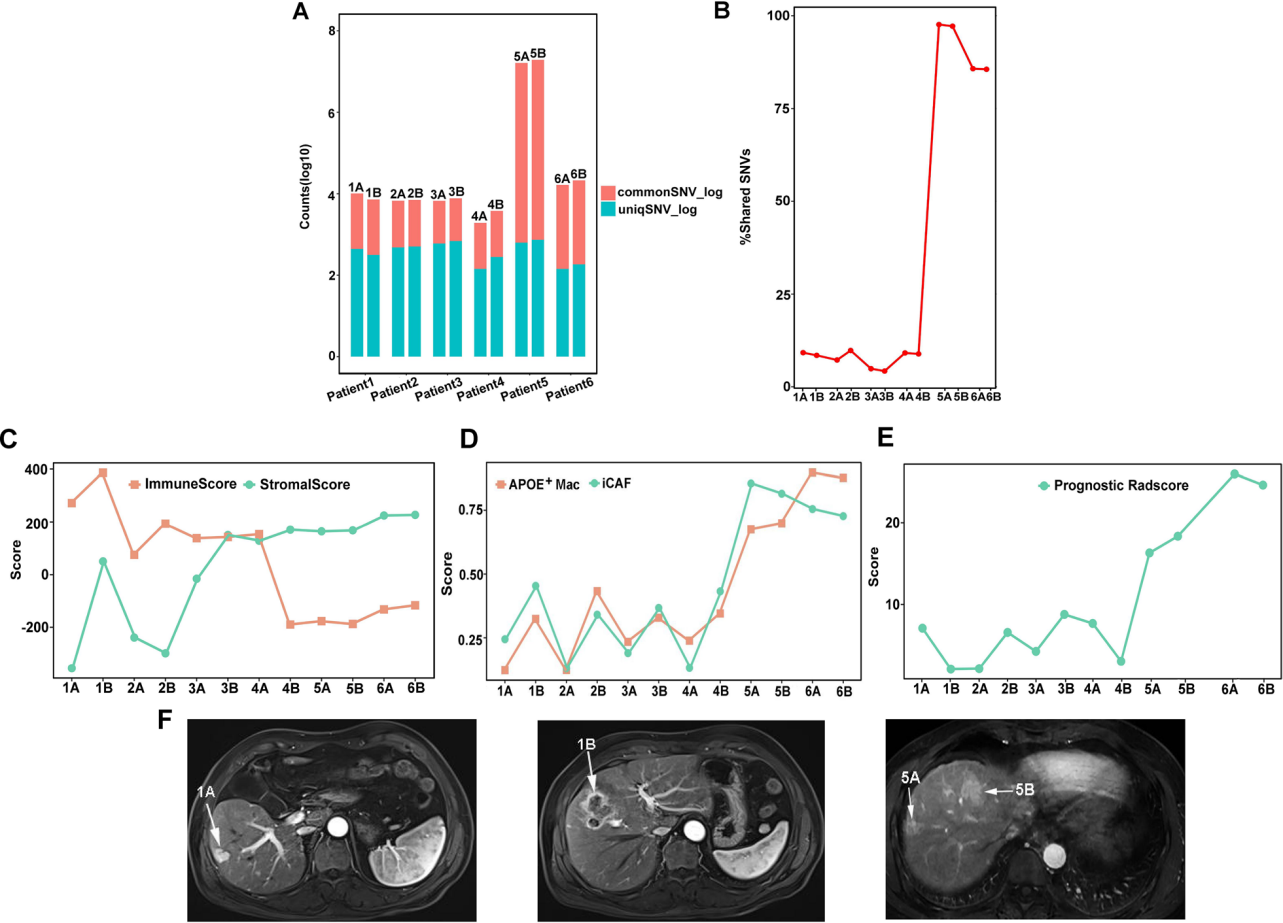
Transcriptome profiles and radiomics score differ between IM and MO. **A** The number of overlapped SNVs and unique SNVs among tumors in six individuals. **B** The proportion of shared SNVs among tumors in six individuals. **C** Line chart showing the level of immune score and stromal score among 12 nodules. **D** Line chart showing the level of APOE^+^ macrophages and iCAFs among 12 nodules. **E** Line chart showing the level of prognostic Radscore among 12 nodules. **F** The MR images of two HCC lesions of patient with MO (Patient 1) showed distinct arterial enhancement pattern while patient with IM (Patient 5) exhibited similar pattern

Since IM was caused by the progression of MVI, we collected 12 tumor samples and 6 adjacent non-tumor liver tissues from 6 patients with bifocal HCC to explore the molecular heterogeneity between IM and MO. Patient 5 and Patient 6 had the presence of MVI. To distinguish IM from MO, we conducted WES analysis to detect mutation rates. We found that IM developed from intrahepatic metastasis with a high rate of common mutations (Patient 5A: 97.63%; Patient 5B: 97.19%; Patient 6A: 85.75%; Patient 6B: 85.59%) while MO patients had minimal shared mutations (Patient 1A: 9.26%; Patient 1B: 8.53%; Patient 2 A: 7.28%; Patient 2B: 9.84%; Patient 3A: 4.94%; Patient 3B: 4.31%; Patient 4 A: 9.18%; Patient 4B: 8.92%) (Fig. 6A, B). To decipher the intratumoral heterogeneity of MO and IM, we observed that MO tumors showed generally lower level of stromal score but higher level of immune score compared with IM tumors (Fig. 6C). Moreover, IM tumors exhibited higher level of APOE^+^ macrophages and iCAFs compared with MO tumors (Fig. 6D). Since it is difficult to achieve differential diagnosis of IM and MO before surgery, radiomics might be an effective tool of displaying differences between them. In our study, prognostic Radscore was highly expressed in IM compared with MO (Fig. 6E) (Table 3). Besides, for MO tumors, two tumors of each patient had more distinct molecular and radiomic differences compared with IM. The MRI of two HCC lesions of Patient 1 also showed distinct arterial enhancement pattern while Patient 5 exhibited similar pattern (Fig. 6F). Our results demonstrated that IM displayed more aggressive tumor behavior than MO, and prognostic Radscore had the potential of distinguishing MO and IM noninvasively.

**Table 3:** Summary of IM/MO diagnosis for 12 HCC nodules from 6 patients

Patient ID	Tumor ID	Synchronous/metachronous	APOE+ Mac	iCAF	ImmuneScore	StromalScore	Prognostic Radscore
Patient1	A	Synchronous	0.123	0.244	271.812	− 355.759	7.089
	B	Synchronous	0.324	0.453	386.329	50.244	2.069
Patient2	A	Synchronous	0.123	0.132	75.109	− 239.102	2.121
	B	Synchronous	0.432	0.340	193.494	− 298.775	6.571
Patient3	A	Synchronous	0.234	0.189	138.356	15.362	4.244
	B	Synchronous	0.328	0.367	142.678	50.254	8.777
Patient4	A	Synchronous	0.238	0.132	− 153.481	178.804	7.671
	B	Synchronous	0.345	0.432	− 189.664	170.685	3.031
Patient5	A	Metachronous	0.675	0.855	− 176.926	164.371	16.313
	B	Metachronous	0.698	0.815	− 187.646	168.088	18.358
Patient6	A	Metachronous	0.897	0.755	− 132.520	224.759	25.968
	B	Metachronous	0.876	0.727	− 116.946	227.215	24.618

## Discussion

MVI is considered as an important risk factor for HCC recurrence after curative resection. Adjuvant therapy may provide survival benefits for HCC patients with MVI [19]. A recent clinical trial showed that adjuvant HAIC with FOLFOX largely improved the disease-free survival benefits in HCC patients with MVI [20]. Our study provided a comprehensive landscape of HCCs with MVI, which might give new clues for exploring novel immunotherapies for HCC patients with MVI.

MVI is likely to progress into IM, Wang et al. reported that IM patients had worse prognoses compared with MO patients after liver transplantation [21]. Dong et al. found that IM had more M2 macrophage and less T cell infiltration [22]. Consistent with our results, we found that IM patients were generally enriched in APOE^+^ macrophages and iCAFs, compared with MO patients. Moreover, the difference of infiltration level between two nodules in IM was relatively small. Above evidences indicate that IM had higher malignancy and less heterogeneity. Since preoperatively distinguishing the clonal origin of multinodular HCC can help select rational liver transplantation candidates to ensure the fair and reasonable use of valuable liver supply resources. Tsuyoshi et al. pointed out that IM had similar CT contrast pattern while MO had heterogeneous CT contrast pattern [23]. However, the ‘similarity’ theory could only be applied in a small group of HCC lesions with significant differences. Therefore, radiomics analysis is a suitable tool to detect the subtle differences of MO lesions. Interestingly, the prognostic radiomics scores we constructed were significantly higher in IM nodules and had minor differences, which has potential to distinguish MO or IM preoperatively. However, our findings should be further validated in a larger cohort.

More and more evidences state that dynamic changes of metabolic state during tumor metastasis adapt to the changing microenvironment [24, 25]. Lipid metabolism is one of the key processes involved in the tumor metastasis. Tumor cells with high metastatic potential express high levels of MAGL, which releases FFA from monoacylglycerol during lipolysis. The uptake of FFA through the FA transporter CD36 increases, which can promote EMT in HCC [26]. Tumor cells not only exhibit increased intake of exogenous lipid but also have high levels of de novo lipogenesis, which leads to aberrant lipid accumulation in the TME. FASN can directly promote the invasion and metastasis of breast cancer cells by mediating the synthesis of fatty acids [27]. Consistent with above findings, we found that MVI positive HCC had aberrantly increased lipid metabolism, which largely depends on the increased level of APOE^+^ macrophages. APOE is a highly specific protein in M2 macrophage-derived exosomes and high expression of APOE tends to be resistant to anti-PD-1 immunotherapy [28, 29]. Tumor-associated macrophages are the most abundant immune cells near the CAFs aggregation region, indicating a close interaction between these two cell types [10, 30]. Qi et al. suggested that SPP1^+^ macrophages stimulate the expression of ECM-related genes in FAP^+^ fibroblasts [31]. iCAFs that are characterized with secreting abundant inflammatory factors like IL-6, IL-8 and IL-11 might participate in tumor metastasis and immune escape [32]. However, the effect of macrophages on iCAFs has not been comprehensively illustrated. We predicted that APOE + macrophages promote the differentiation of iCAF through the SPP1/CD44 interaction.

The evaluation of TME relies almost entirely on pathological methods, and the invasive nature of the methods limits the wider application of TME evaluation. Considering the complex component of TME, previous studies has established multiple reliable radiomics models to predict immune cells in TME. Yoon et al. demonstrated the feasibility of using radiomics model to predict the infiltration of Th2 cells in non-small cell lung cancer [33]. Khorrami et al. found that radiomics features derived from peritumoral non-small cell lung cancer can effectively predict the tumor-infiltrating lymphocytes [34]. With the rise of scRNA-seq, more diverse cell types and their interactions in TME have been found. Interestingly, in our study, the MVI prediction radiomics model can also effectively predict the proportion of APOE^+^ macrophages and iCAFs. Moreover, the prognostic radiomics model was highly correlated with the level of APOE^+^ macrophages and iCAFs. Such findings provide opportunities of evaluating TME noninvasively and efficiently.

There are several limitations of our study. First, the retrospective nature of our study might introduce selection bias because we only included surgical patients. Second, the size of transcriptomic cohort was relatively small. The generalizability of radiomics features for predicting the proportion of TME should be further validated in larger radiotranscriptomic cohort. Third, clinical validation of our results in HCC patients with MVI in immunotherapy cohort should be conducted.

In conclusion, our study reveals changes in the TME of MVI positive patients, which provides more detailed information for new adjuvant treatment. Special attention can be paid to reducing lipid metabolism in MVI positive patients. Besides, our research links radiomics with TME, thereby achieving non-invasive evaluation of tumor microenvironment components, especially during the treatment process, which can optimize patient immunotherapy plans, and reduce the recurrence rate of MVI positive patients.

### Supplementary Information

**Additional file 1: Table S1.** The sequences of primers. **Table S2.** Optimal features in the MVI prediction radiomics model. **Table S3.** Summary of radiomics scores in 6 HCC patients with scRNA-seq data. **Table S4.** Optimal features in the HCC prognostic radiomics model. **Figure S1.** scRNA-seq profiling of T cells in MVI^+^ HCC and MVI^−^ HCC. (A) tSNE showing the composition of CD8^+^ T cells. (B) Bar plots showing the percentage of three CD8^+^ T cells subtypes. (C) tSNE showing the composition of CD4^+^ T cells. (D) Bar plots showing the percentage of three CD4^+^ T cells subtypes. (E) Scatter plots showing the correlation between the infiltration of APOE^+^ macrophages and CD8_Effector T cells. (F) Scatter plots showing the correlation between the infiltration of APOE^+^ macrophages and CD4_Treg. **Figure S2.** (A) The infiltration of CD4_Treg calculated by CIBERSORTX in different HLF expression groups in ZS cohort. (B) Fatty acid metabolism score calculated by GSVA in different HLF expression groups in Zhongshan cohort. **Figure S3.** (A) APOE mRNA level in THP-1 derived macrophages 48h after transfection of APOE shRNA. (B)The mRNA expression level of the four metastasis-related gene in Huh7 cells when were co-cultured with THP-1 derived macrophages or shAPOE THP-1 derived macrophages. An unpaired two-tailed t-test was applied. **Figure S4.** Dotplot showing upregulated and downregulated interactions among APOE^+^ macrophages, iCAFs and CD8^+^ T cells. **Figure S5.** Validation of radiomic scores in multiple clinical cohorts. (A) AUC of the MVI prediction Radscore for predicting MVI in the training cohort, validation cohort A and validation cohort B. (B) Kaplan–Meier plots show the prognostic value of prognostic Radscore for overall survival in the training cohort, validation cohort A and validation cohort B.

## Data Availability

The data that support the findings of this study are available from the corresponding author upon reasonable request.

## Abbreviations

HCC: Hepatocellular carcinoma
MVI: Microvascular invasion
APOE: Apolipoprotein E
GSVA: Gene set variation analysis
HLF: Hepatic leukaemia factor
iCAFs: Inflammatory CAFs
ROC: Receiver operator characteristics
CAF: Cancer-associated fibroblast
WES: Whole Exome Sequencing
IM: Intrahepatic metastasis
MO: Multicentric occurrence
TME: Tumor microenvironment
LASSO: Least absolute shrinkage and selection operator
ROC: Receiver operator characteristics
