# Supplementary material for: Deciphering intratumoral heterogeneity of hepatocellular carcinoma with microvascular invasion with radiogenomic analysis
Source: J Transl Med. 2023 Oct 18;21:734. doi: 10.1186/s12967-023-04586-6 (PMC10583459; doi:10.1186/s12967-023-04586-6)
Supplement: Supplementary file 1 — Additional file 1: Table S1. The sequences of primers. Table S2. Optimal features in the MVI prediction radiomics model. Table S3. Summary of radiomics scores in 6 HCC patients with scRNA-seq data. Table S4. Optimal features in the HCC prognostic radiomics model. Figure S1. scRNA-seq profiling of T cells in MVI+ HCC and MVI− HCC. (A) tSNE showing the composition of CD8+ T cells. (B) Bar plots showing the percentage of three CD8+ T cells subtypes. (C) tSNE showing the composition of CD4+ T cells. (D) Bar plots showing the percentage of three CD4+ T cells subtypes. (E) Scatter plots showing the correlation between the infiltration of APOE+ macrophages and CD8_Effector T cells. (F) Scatter plots showing the correlation between the infiltration of APOE+ macrophages and CD4_Treg. Figure S2. (A) The infiltration of CD4_Treg calculated by CIBERSORTX in different HLF expression groups in ZS cohort. (B) Fatty acid metabolism score calculated by GSVA in different HLF expression groups in Zhongshan cohort. Figure S3. (A) APOE mRNA level in THP-1 derived macrophages 48h after transfection of APOE shRNA. (B)The mRNA expression level of the four metastasis-related gene in Huh7 cells when were co-cultured with THP-1 derived macrophages or shAPOE THP-1 derived macrophages. An unpaired two-tailed t-test was applied. Figure S4. Dotplot showing upregulated and downregulated interactions among APOE+ macrophages, iCAFs and CD8+ T cells. Figure S5. Validation of radiomic scores in multiple clinical cohorts. (A) AUC of the MVI prediction Radscore for predicting MVI in the training cohort, validation cohort A and validation cohort B. (B) Kaplan–Meier plots show the prognostic value of prognostic Radscore for overall survival in the training cohort, validation cohort A and validation cohort B. [file 12967_2023_4586_MOESM1_ESM.docx]

**Appendices**

**Table S1. The sequences of primers**

|  | Sense 5’-3’ | Antisense 5’-3’ |
| --- | --- | --- |
| APOE | GTTGCTGGTCACATTCCTGG | GCAGGTAATCCCAAAAGCGAC |
| VEGFA | AGGGCAGAATCATCACGAAGT | AGGGTCTCGATTGGATGGCA |
| CDH1 | CGAGAGCTACACGTTCACGG | GGGTGTCGAGGGAAAAATAGG |
| MMP-2 | GATACCCCTTTGACGGTAAGGA | CCTTCTCCCAAGGTCCATAGC |
| MMP-9 | TGTACCGCTATGGTTACACTCG | GGCAGGGACAGTTGCTTCT |

**Table S2. Optimal features in the MVI prediction radiomics model**

| Radiomic feature | Coefficient |
| --- | --- |
| log_gldm_log-sigma-1-5-mm-3d-smalldependencelowgraylevelemphasis_AP | -1.199 |
| wavelet_glszm_wavelet-hll-smallarealowgraylevelemphasis_AP | -0.337 |
| log_glrlm_log-sigma-0-5-mm-3d-shortrunlowgraylevelemphasis_PVP | -0.900 |
| original_shape_surfacevolumeratio_PVP | 0.065 |
| log_gldm_log-sigma-0-5-mm-3d-smalldependencelowgraylevelemphasis_PVP | -0.348 |
| log_glrlm_log-sigma-0-5-mm-3d-longrunlowgraylevelemphasis_DP | 0.284 |

AP, arterial phase; PVP, portal venous phase; DP, delayed phase

**Table S3. Summary of radiomics scores in 6 HCC patients with scRNA-seq data.**

|  | MVI Radscore | MVI state | Prognostic Radscore | APOE+Mac | iCAF |
| --- | --- | --- | --- | --- | --- |
| Patient A | 8.34 | MVI+ | 9.21 | 30.8% | 9.3% |
| Patient B | 6.75 | MVI+ | 8.39 | 35.8% | 17.4% |
| Patient C | 7.23 | MVI+ | 5.98 | 33.1% | 14.7% |
| Patient D | 3.45 | MVI- | 1.34 | 30.7% | 6.0% |
| Patient E | 5.14 | MVI- | 3.45 | 10.2% | 51.8% |
| Patient F | 1.34 | MVI- | 2.65 | 25.6% | 10.9% |

**Table S4. Optimal features in the HCC prognostic radiomics model**

| Radiomic feature | Coefficient |
| --- | --- |
| boxsigmaimage_glcm_imc1_AP | -0.397 |
| boxsigmaimage_glrlm_runvariance_AP | 0.141 |
| wavelet_firstorder_wavelet-lhh-skewness_AP | 0.299 |
| wavelet_firstorder_wavelet-hll-median_AP | 0.124 |
| wavelet_firstorder_wavelet-hlh-totalenergy_AP | 0.155 |
| wavelet_firstorder_wavelet-hhh-uniformity_AP | 0.253 |
| wavelet_glcm_wavelet-hll-differencevariance_PVP | 0.165 |
| wavelet_glrlm_wavelet-hhh-lowgraylevelrunemphasis_PVP | 0.138 |
| wavelet_glszm_wavelet-lhl-lowgraylevelzoneemphasis_PVP | 0.236 |
| wavelet_glszm_wavelet-lhl-smallarealowgraylevelemphasis_PVP | 0.316 |
| wavelet_glszm_wavelet-lhh-sizezonenonuniformitynormalized_PVP | 0.391 |
| wavelet_glszm_wavelet-hlh-smallareahighgraylevelemphasis_PVP | 0.264 |
| wavelet_glszm_wavelet-hlh-zoneentropy_DP | 0.028 |
| wavelet_glszm_wavelet-hhh-smallareaemphasis_DP | 0.250 |
| wavelet_gldm_wavelet-hhl-graylevelvariance_DP | 0.143 |
| specklenoise_firstorder_minimum_DP | -8.894 |

AP, arterial phase; PVP, portal venous phase; DP, delayed phase

**
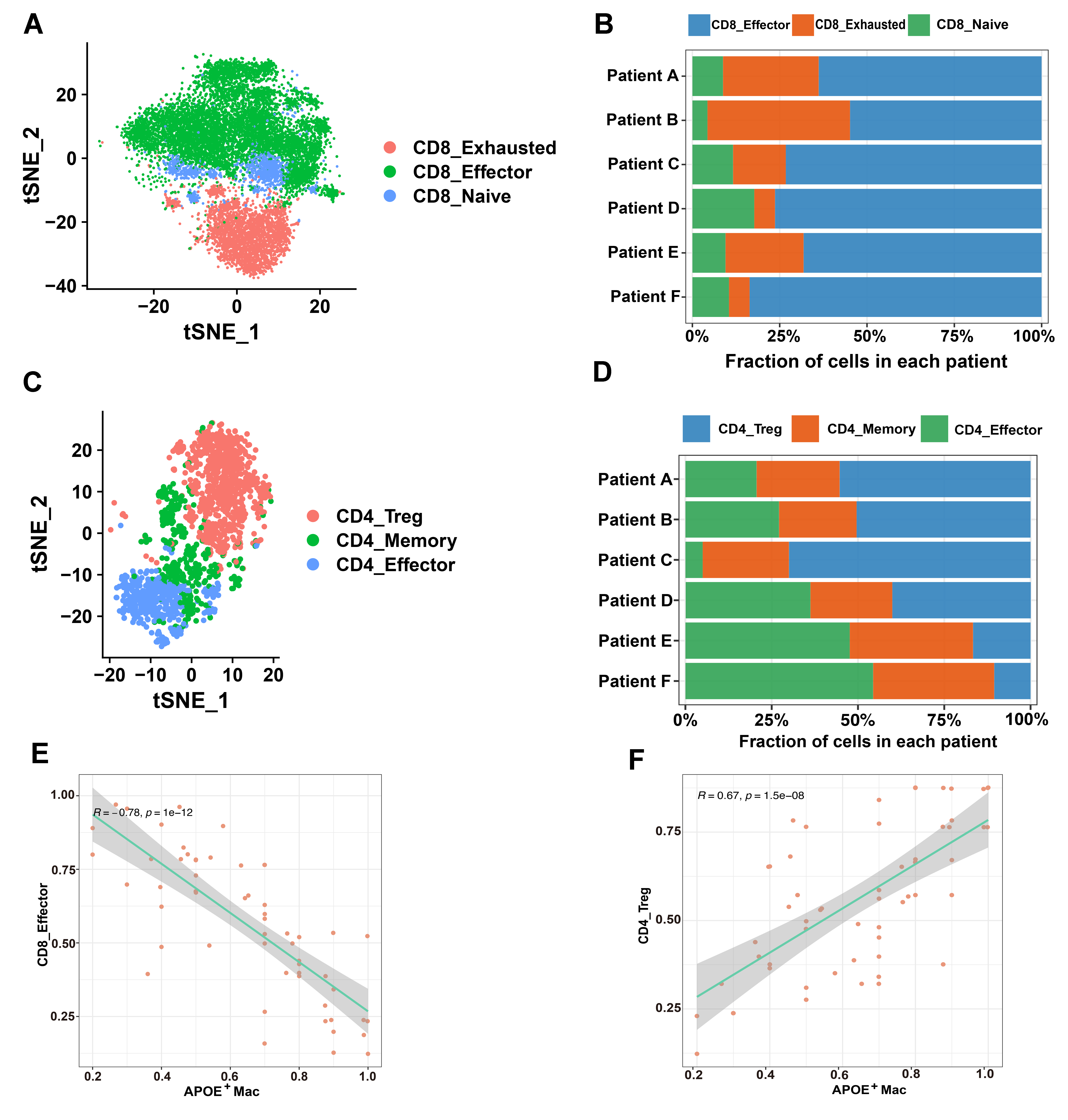
Figure S1**: scRNA-seq profiling of T cells in MVI^+^ HCC and MVI^-^ HCC. (A) tSNE showing the composition of CD8^+^ T cells. (B) Bar plots showing the percentage of three CD8^+^ T cells subtypes. (C) tSNE showing the composition of CD4^+^ T cells. (D) Bar plots showing the percentage of three CD4^+^ T cells subtypes. (E) Scatter plots showing the correlation between the infiltration of APOE^+^ macrophages and CD8_Effector T cells. (F) Scatter plots showing the correlation between the infiltration of APOE^+^ macrophages and CD4_Treg.

**
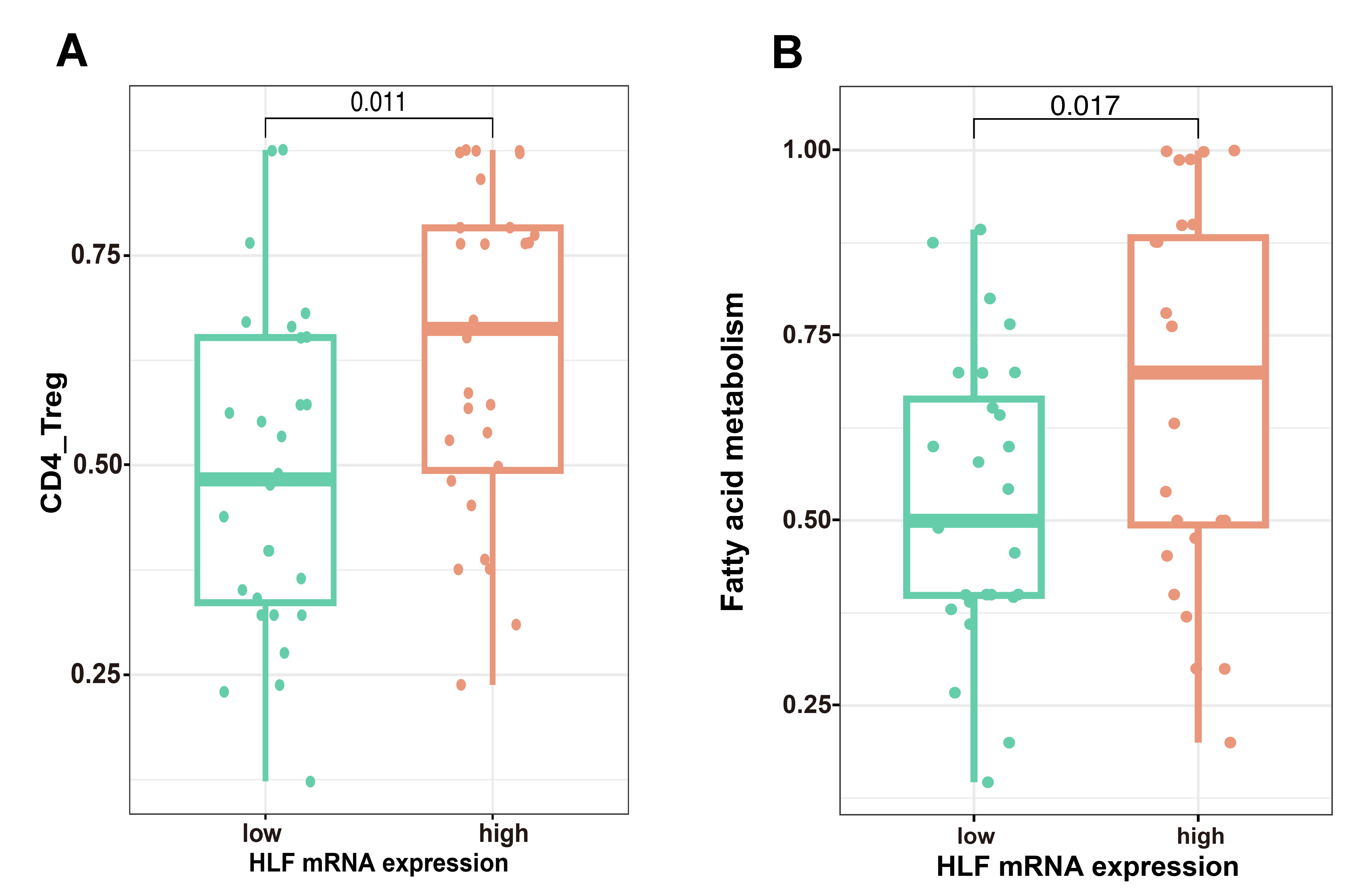
Figure S2:** (A) The infiltration of CD4_Treg calculated by CIBERSORTX in different HLF expression groups in ZS cohort. (B) Fatty acid metabolism score calculated by GSVA in different HLF expression groups in Zhongshan cohort.

**
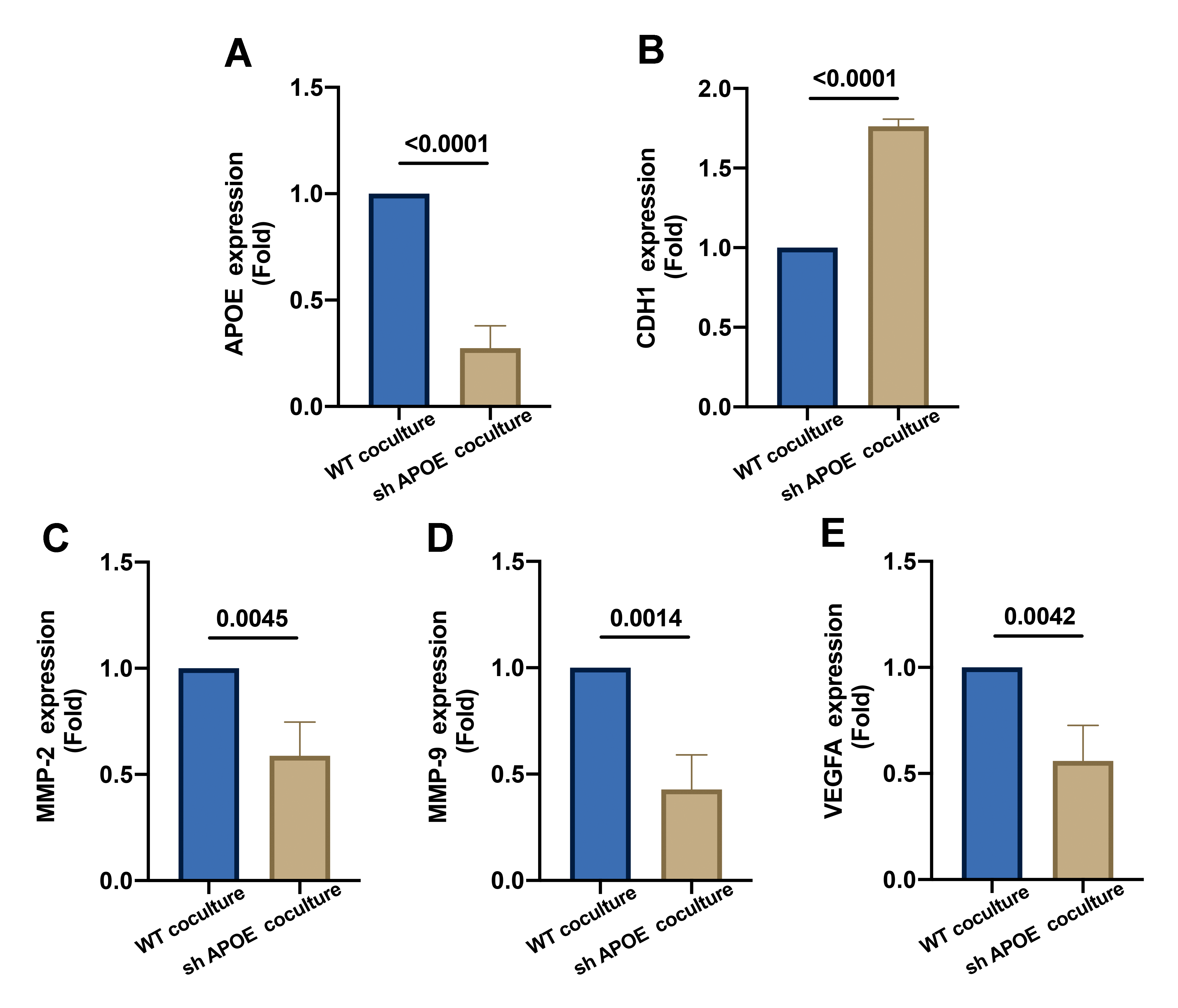
Figure S3:** (A) APOE mRNA level in THP-1 derived macrophages 48h after transfection of APOE shRNA. (B)The mRNA expression level of the four metastasis-related gene in Huh7 cells when were co-cultured with THP-1 derived macrophages or shAPOE THP-1 derived macrophages. An unpaired two-tailed t-test was applied.

**
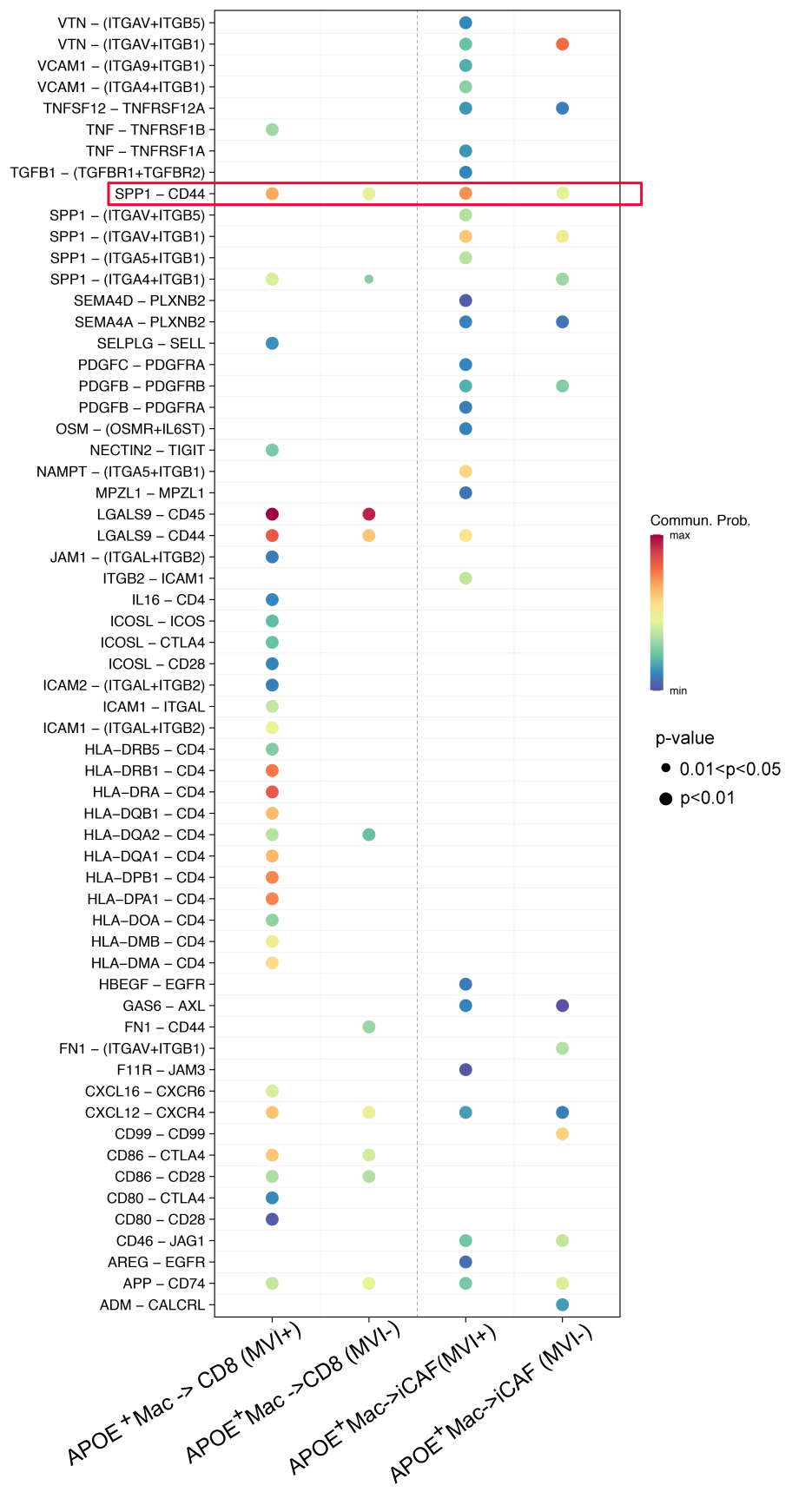
**

**Figure S4:** Dotplot showing upregulated and downregulated interactions among APOE^+^ macrophages, iCAFs and CD8^+^T cells.

**
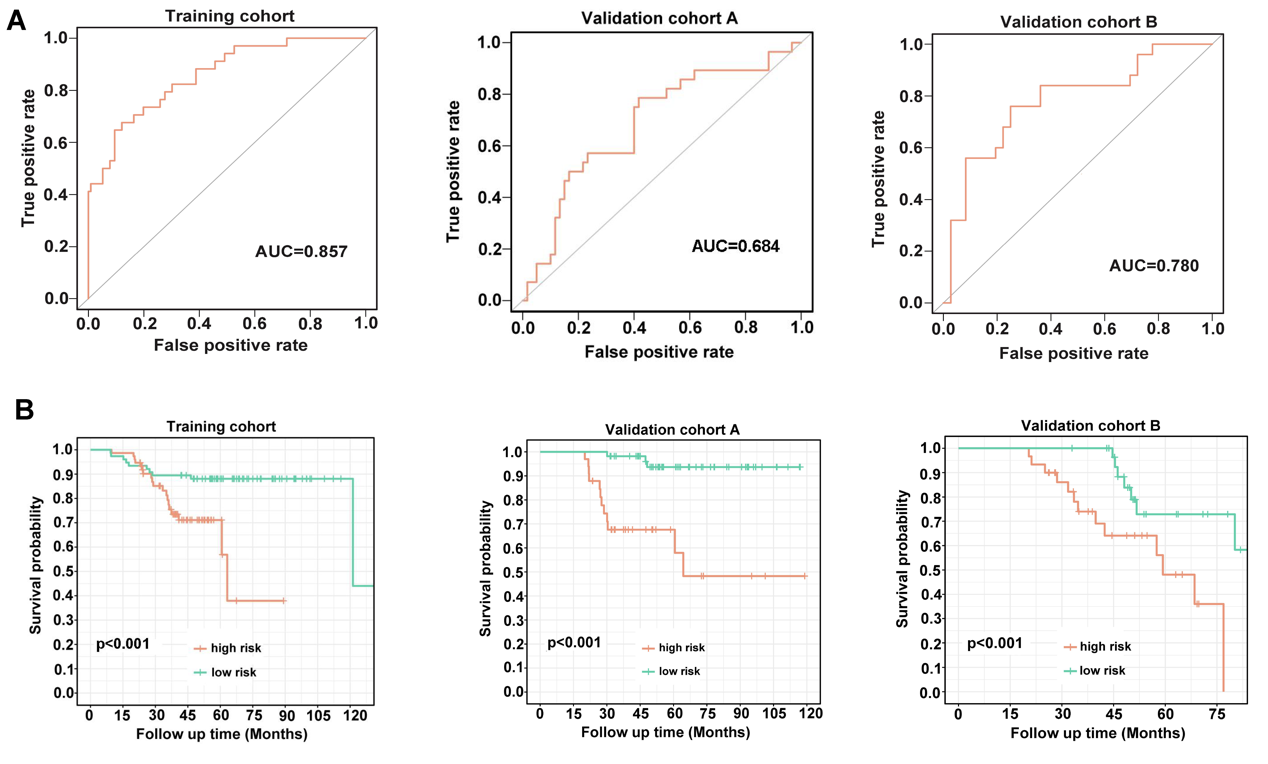
**

**Figure S5.** Validation of radiomic scores in multiple clinical cohorts. (A) AUC of the MVI prediction Radscore for predicting MVI in the training cohort, validation cohort A and validation cohort B. (B) Kaplan-Meier plots show the prognostic value of prognostic Radscore for overall survival in the training cohort, validation cohort A and validation cohort B.
